# Responses of the Hybrid between *Sphagneticola trilobata* and S*phagneticola calendulacea* to Low Temperature and Weak Light Characteristic in South China

**DOI:** 10.1038/srep16906

**Published:** 2015-11-19

**Authors:** Zhongyu Sun, Yanqiao Chen, Valentin Schaefer, Huimiao Liang, Weihua Li, Shengqin Huang, Changlian Peng

**Affiliations:** 1 Guangzhou Key Laboratory of Subtropical Biodiversity and Biomonitor, Guangdong Provincial Key Laboratory of Biotechnology for Plant Development, School of Life Sciences, South China Normal University, Guangzhou 510631, China; 2 Guangdong Open Laboratory of Geospatial Information Technology and Application, Guangzhou Institute of Geography, Guangzhou 510070, China; 3 School of Environmental Studies, Faculty of Social Sciences, University of Victoria, Victoria, BC V8W 5Y2, Canada

## Abstract

Hybridization between exotic and native species is of great interest to evolutionary biologists and ecologists because it usually shows a quick evolution of species and invasiveness. It has been reported that such hybridization frequently increases the adaptation and aggressiveness of the new hybrids. A hybrid between invasive *Sphagneticola trilobata* and its native congener *S. calendulacea* was recently found in subtropical China. S*. calendulacea* has a significantly higher tolerance to low temperature and weak light stress than *S. trilobata*, and its range includes both tropical and temperate regions. This study examined how the tolerance of the new hybrid to low temperature and weak light conditions (LTWL), expanded its geographical range. All changes of phenotype, gas exchange parameters, chlorophyll fluorescence parameters, contents of malonaldehyde (MDA) and activity of superoxide dismutase (SOD) and peroxidase (POD) indicated that hybridization slightly catalyzed the tolerance of the hybrid to LTWL condition and the responses of the hybrid were more similar with their invasive parent. The results demonstrate that the current hybrid populations may not expand their geographical distribution ranges in a short period, but the distribution of the backcrossed generations is still uncertain. The threat of the hybrid to its native parent in subtropical region should be concerned.

Hybridization between species is commonplace in plants. At least 25% of plant species hybridize and potentially can introgress with other species^1^. Hybridization between introduced and native plant species is also common^2^,^3^,^4^,^5^. Hybridization between native and invasive species is one of the primary drivers behind the evolution of invasiveness, considered the second most important threat to biodiversity after habitat degradation^6^,^7^,^8^. The increase in invasiveness of hybrids is probably due to evolutionary novelty, genetic variation, fixed heterosis, and reduced genetic load^9^. As a result, hybrids between native and invasive plant taxa usually demonstrate greater fitness and wider ecological tolerances than their parents^8^,^9^. Such hybrids can harm native species through the loss of both genetic diversity and of locally adapted populations. Aggressive hybrid taxa can also result in reduced growth and even the extirpation of native species populations^10^,^11^. However, it is not yet possible to predict which of the hybridizations will lead to stable zones and hybrid swarms^12^, because evolutionary invasiveness is closely related to the genetic distance between mating colonists; not all hybridizations will lead to the evolution of invasiveness^9^.

*Sphagneticola trilobata* (L.) Pruski (synonyme: *Wedelia trilobata*, *S. trilobata* hereafter) (2n = 4 ×= 56, Harriman^13^) is a native and creeping perennial herb in South America. It is now widespread in tropical and subtropical regions around the world. Recently, it has been considered to be one of the IUCN’s 100 most invasive species^14^. In the 1970’s, *S. trilobata* was introduced into Southern China as a groundcover for its fast growth and hardiness. However, it rapidly escaped from cultivation and became invasive. Escaped *S. trilobata* crowds out native species in communities through competition for nutrients, light and water, and usually forms a monoculture in natural areas or plantations^15^,^16^. There is a native Chinese congener of *S. trilobata*, i.e., S*phagneticola calendulacea* (L.) Pruski (synonyme: *Wedelia chinensis* Merr, *S. calendulacea* hereafter) (2n = 4 ×= 50, Mehra and Remanandan^17^). It has greater tolerance to cold, ranging from subtropical China to temperate areas^18^. *S. trilobata* and its native relative *S. calendulacea* usually occur together along riverbanks, coastlands and other moist habitats in South China. With the rapid expansion of *S. trilobata* during the last two decades in South China, the distribution and abundance of native *S. calendulacea* have decreased significantly^19^. *S. trilobata* has a higher photosynthetic rate and lower construction cost than its native relative *S. calendulacea*^15^,^20^,^21^. Some reports in China argue that *S. trilobata* could improve soil quality, including an increase in soil organic matter, total nitrogen, available phosphorus and available potassium, as well improving the community of soil microbes^22^,^23^. These changes create a habitat suitable for *S. trilobata* and enhance its invasiveness.

A species with a phenotype between *S. trilobata* and *S. calendulacea* was found in the wild and identified as the hybrid between *S. trilobata* × *S. calendulacea* in 2013^19^. Ni *et al.*^24^ suggested that the hybrid was an equal competitor to its invasive parent and was more susceptible to N deposition under N deposition treatments. Nevertheless, little has been reported about its tolerance to cold and weak light conditions, information necessary in both managing invasions and understanding the ecological and evolutionary aspects of *S. trilobata, S. calendulacea* and their hybrid.

We designed an experiment to assess the tolerance of the hybrid between *S. trilobata* and *S. calendulacea* to extreme winter conditions (low temperature and weak light, LTWL) in subtropical China. We addressed: (1) The response of the hybrid to LTWL stress. (2) The evolution of tolerance to LTWL stress by hybrids.

## Results

### The phenotypic change

**Figure 1:**
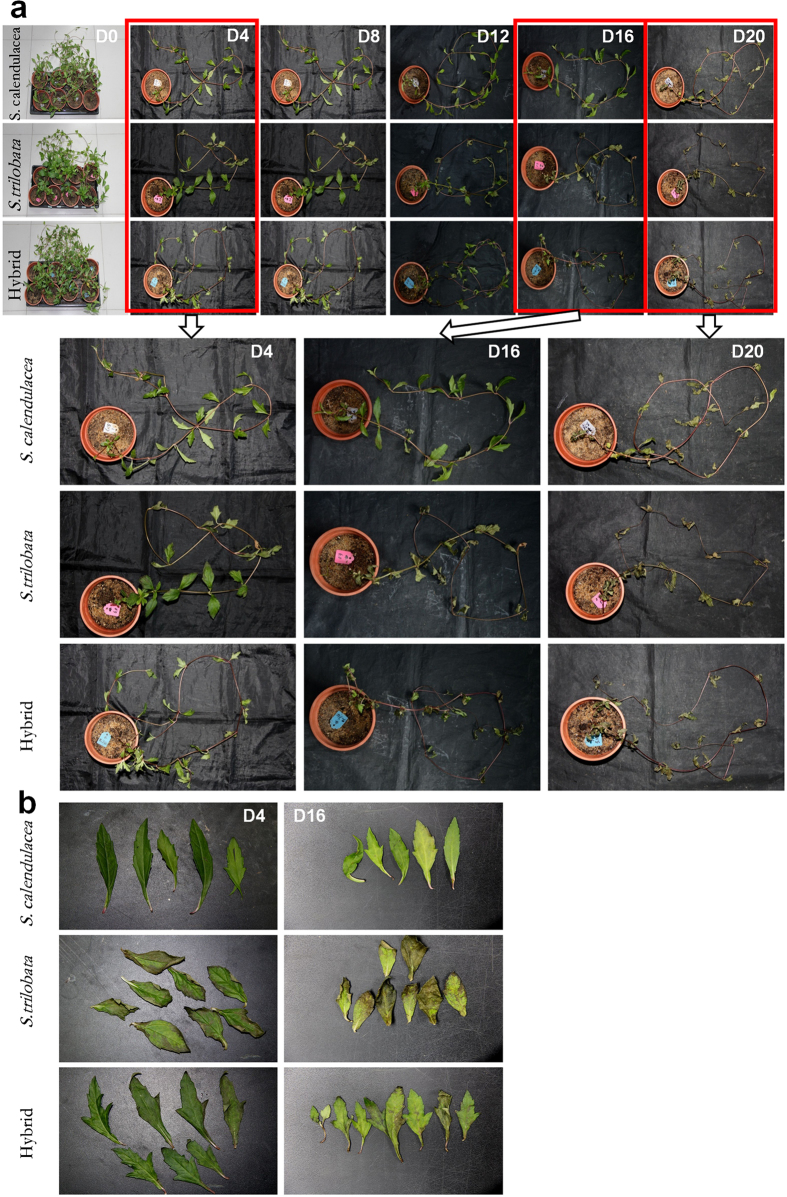
The phenotypic change and leaves-change of *S. trilobata*, *S. calendulacea* and their hybrid during the cold and weak light treatment. D0–D20 means 0–20 days after treatment.

After four days, all species showed some damage. The edges of leaves of the invasive *S. trilobata* were withered and yellow. After 16 days of treatment, the phenotypic changes of these three species were obviously different (Fig. 1a). The leaves of hybrids were wilted and largely damaged by the LTWL treatment. The leaves of the invasive species were damaged more heavily and plants almost died. In contrast, the leaves of native species were still fresh and green and showed little damage (Fig. 1b). After 20 days of treatment, both hybrid and invasive species died while some leaves of the native species survived. The results of phenotypic change indicated that the tolerance of the hybrid to LTWL was intermediate between that of its parents.

### The change of gas exchange parameters

**Figure 2:**
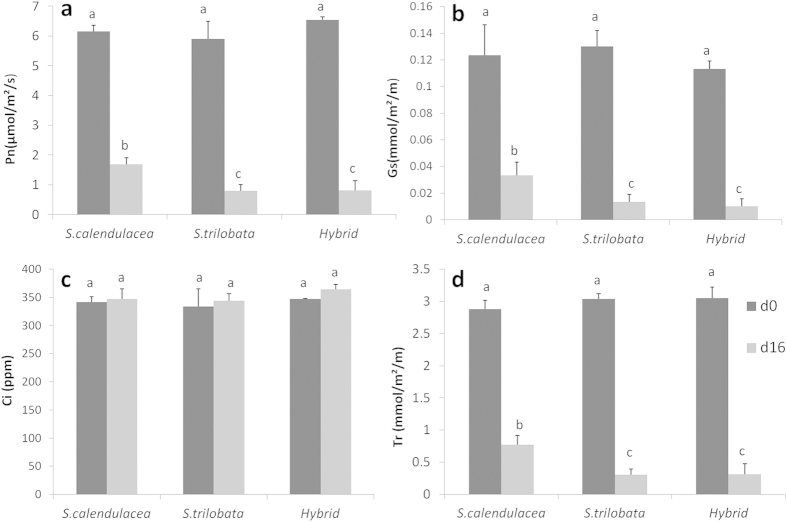
Pn, Gs, Ci and Tr of *S. trilobata*, *S. calendulacea* and their hybrid before (d0) and after 16 days (d16) of treatment. The different letters between the columns means significantly different (one way ANOVA, n = 4, LSD, P = 0.05).

**Figure 3:**
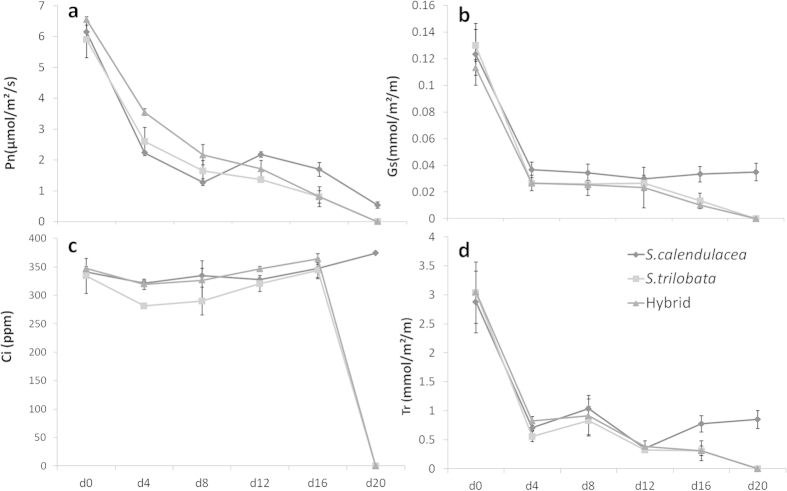
The continous observations of Pn, Gs, Ci and Tr of *S. trilobata*, *S. calendulacea* and their hybrid during the treatment.

The net photosynthetic rate (Pn), stomatal conductance (Gs), and transpiration rate (Tr) of *S*. *trilobata*, *S. calendulacea* and their hybrid before (d0) and after 16 days (d16) of treatment are shown in Fig. 2. Before the treatment, Pn, Gs and Tr of the hybrid were not significantly different from the parents. After 16 days of treatment, Pn, Gs and Tr of all three decreased significantly. Pn, Gs and Tr of the hybrid was significantly lower than its native parent, but not different from its invasive parent. The continuous observations data (Fig. 3) shows that Pn of *S. calendulacea* decreased quickly in the first 8 days, but increased on day-12 and then deceased until death. After 12 days of treatment, *S. calendulacea* showed obviously higher Gs and Tr than *S. trilobata* and the hybrid. During 20 days of treatment, the responses of the hybrid were always similar to its invasive parent *S. trilobata*. The gas exchange parameters indicate that the tolerance of the hybrid to LTWL was significantly weaker than its native parent *S. calendulacea* but not significantly different from its invasive parent *S. trilobata*.

### The change of chlorophyll fluorescence parameters

**Figure 4:**
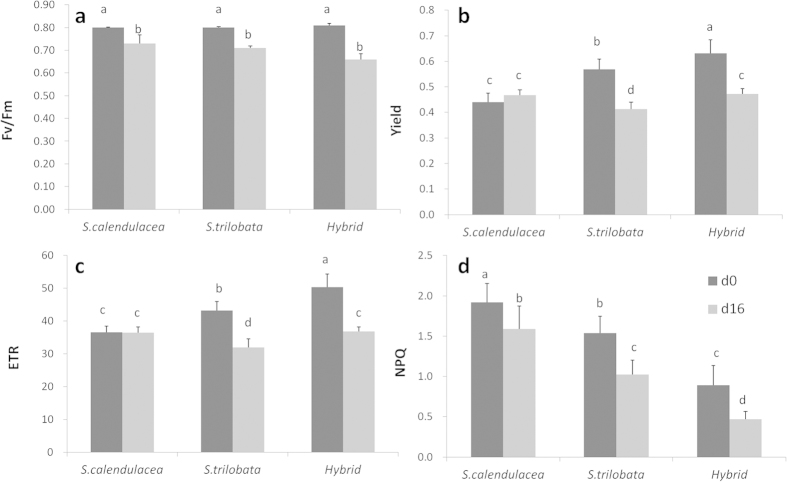
F_v_/F_m_, Yield ETR and NPQ of *S. trilobata*, *S. calendulacea* and their hybrid before (d0) and after 16 days (d16) of treatment. The different letters between the columns means significantly different (one way ANOVA, n = 4, LSD, P = 0.05).

**Figure 5:**
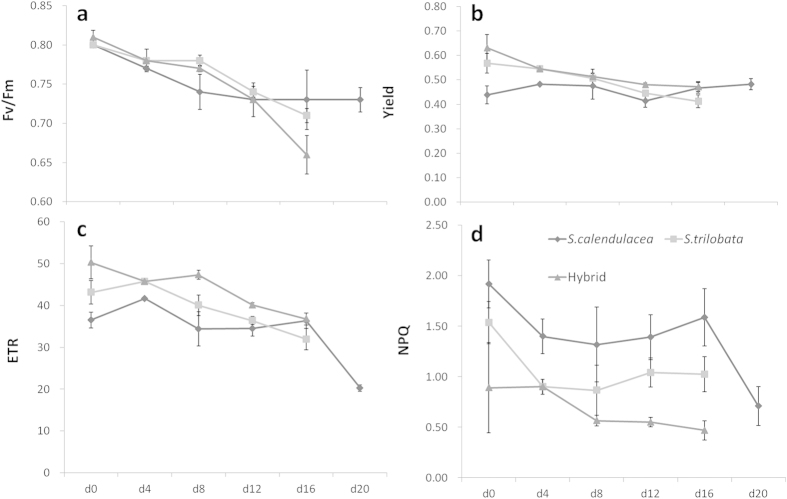
The continous observations of F_v_/F_m_, Yield, ETR and NPQ of *S. trilobata*, *S. calendulacea* and their hybrid during the treatment.

Before treatment, values of F_v_/F_m_ were not significantly different between the two species. However, both Yield and ETR of the hybrid were significantly higher than their parents. After 16 days of treatment, the value of F_v_/F_m_ of all species decreased significantly (Fig. 4a). The decreases in Yields and ETRs of the hybrid and *S. trilobata* were significant while there was no significant change for *S. calendulacea*. The continuous observations (Fig. 5) showed that F_v_/F_m_, Yield and ETR of these three species all decreased during the treatment. From beginning to end, NPQ of the native *S. calendulacea* was always higher than invasive *S. trilobata* and the hybrid.

**Figure 6:**
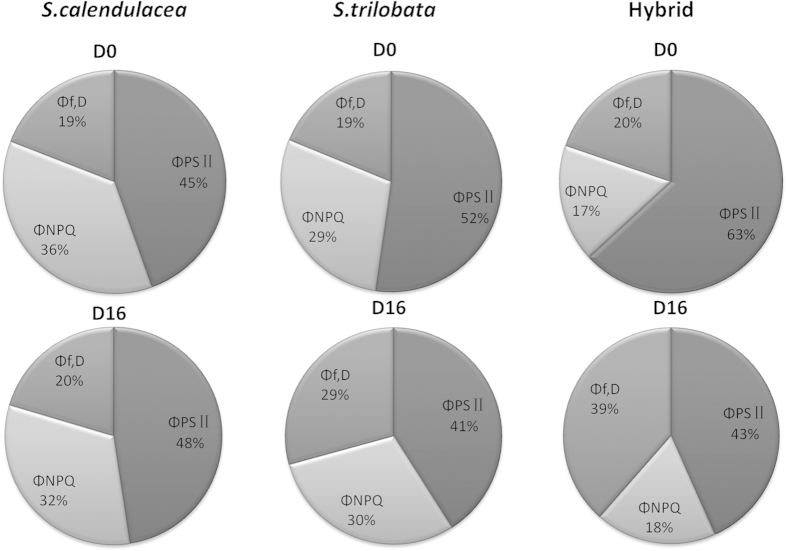
The allocation of absorbed light energy in leaves of *S. trilobata*, *S. calendulacea* and their hybrid before and after 16 days of treatment.

The allocations of absorbed light energy in leaves of *S. trilobata*, *S. calendulacea* and their hybrid before and after 16 days of treatment are shown in Fig. 6. Before treatment, the proportion of Φ_PS II_ of hybrid (63%) was obviously higher than that of native (45%) and invasive species (45%), while the proportion of Φ_NPQ_ (17%) was much lower than its parents (36% and 29%). However, the proportion of Φ_f,D_ was little different between all three. After 16 days of treatment, the proportions of Φ_PS II_ of hybrid and invasive species decreased sharply (63% to 43% and 52% to 41%), while their Φ_f,D_ proportions largely increased (20% to 39% and 19% to 29%) and the proportion of Φ_NPQ_ had little change. At the same time, the proportions of Φ_PS II_, Φ_NPQ_, and Φ_f,D_ of native species showed little change after 16 days of treatment.

### The changes of malonaldehyde (MDA), superoxide dismutase (SOD) and peroxidase (POD)

**Figure 7:**
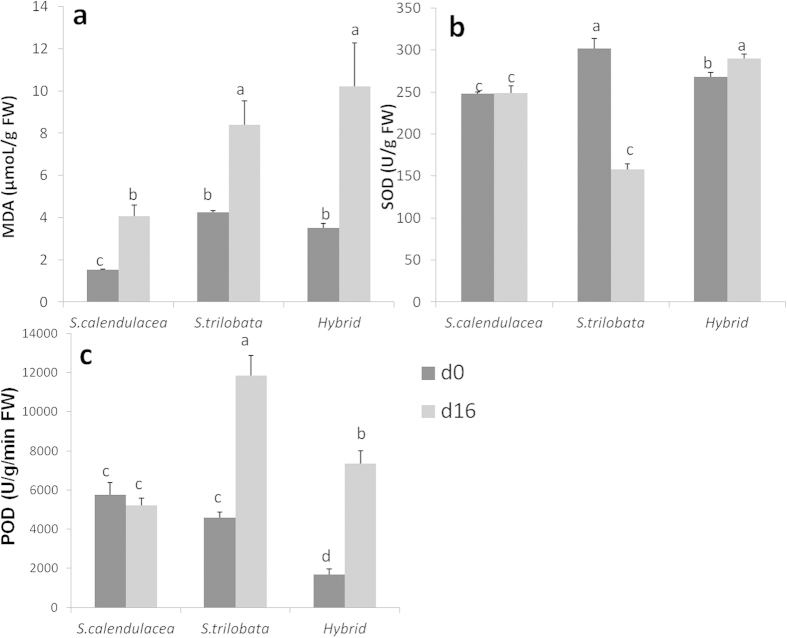
The change of MDA, SOD and POD in leaves of *S. trilobata*, *S. calendulacea* and their hybrid before (d0) and after 16 days (d16) of treatment. The different letters between the columns means significantly different (one way ANOVA, n = 4, LSD, P = 0.05).

**Figure 8:**
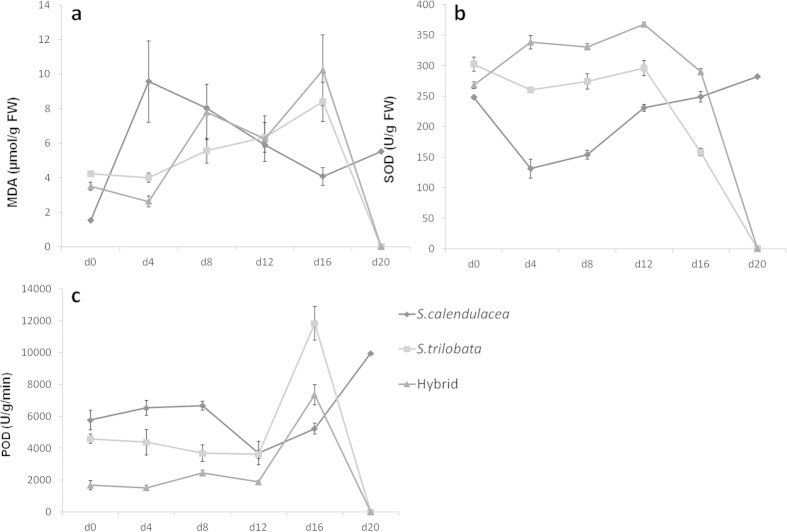
The continous observations of MDA, SOD and POD in leaves of *S. trilobata*, *S. calendulacea* and their hybrid during the treatment.

Before treatment, a small amount of MDA existed in all species (Fig. 7a). The activity of SOD of the hybrid was intermediate between their parents. After 16 days of treatment, the content of MDA of all species increased significantly, especially the hybrid. The activity of SOD of hybrid significantly increased after treatment, while that of the invasive species *S*. *trilobata* significantly decreased (Fig. 7b). At the same time, the activity of SOD of *S. calendulacea* was little changed. After 16 days of treatment, the activity of POD in the hybrid increased by 338.17%, *S*. *trilobata* increased 157.97% and *S. calendulacea* had little change. The continuous observations showed that the content of MDA for *S. calendulacea* increased sharply in the first 4 days and then decreased until the end of treatment, but for *S*. *trilobata* and the hybrid it increased from beginning to end (Fig. 8). The results for MDA, SOD and POD indicate that the tolerance of the hybrid to low temperature and low light was little better than the invasive species but significantly weaker than the native species.

### Discussion

This research indicates that hybridization slightly catalyzes the tolerance of the hybrid to LTWL conditions and the responses of the hybrid to LTWL were more similar to that of its invasive parent *S. trilobata.* It has been reported that *S. trilobata* was more sensitive to LTWL than its native congener *S. calendulacea*^25^,^26^, but for the new hybrid, its tolerance is still unclear. Wu *et al.* (2013)^19^ found that the hybrid showed slightly lower or equivalent growth potential compared to its invasive parent *S. trilobata* but significantly higher energy-use efficiency than both native and invasive parents based on the studies of their photosynthesis, specific leaf area (SLA), leaf nutrient concentration, and construction cost. The hybrid has also been found to be an equal competitor to its invasive parent but was more susceptible to N deposition^24^. Our results are also consistent with previous studies on the hybrid between *S. trilobata* and *S. calendulacea*. In this study, the result of phenotypic change indicated that the tolerance of the hybrid to LTWL seemed intermediate between that of its parents. After 16 days of treatment, Pn, Gs, and Tr of the hybrid were not significantly different from its invasive parent *S. trilobata*, but significantly lower than its native parent *S. calendulacea.* These changes of gas exchange parameters indicate that the hybrid dose not demonstrate heterosis on resistance to LTWL condition. The relative increment of yield, ETR and the activity of SOD and POD of the hybrid were significantly higher than its invasive parent *S. trilobata*, although significantly lower than its native parent *S. calendulacea.* Higher increased activity of SOD and POD (Fig. 7) of the hybrid after 16 days of treatment contributes to scavenging O_2_^−^ and enhances the tolerance of the hybrid to LTWL condition. To some degree, this result explains the phenotypic differences between the hybrid and *S. trilobata* under LTWL treatment. All changes in phenotype, gas exchange parameters, chlorophyll fluorescence parameters, contents of MDA and activity of SOD and POD indicate that the responses of the hybrid to LTWL were very similar to its invasive parent *S. trilobata.*

This phenomenon has also been found in the hybrid between invasive *Carpobrotus edulis* and its native congener *C. chilensis*^27^. The hybrids are very similar in their growth and salt tolerance to the introduced taxon *Carpobrotus edulis*^27^. In addition, the continuous observations show that the Pn of *S. calendulacea* have a significant recovery on day-12 (Fig. 3a). It may be relative to the stronger adaptability or resilience of *S. calendulacea* to LTWL condition. The activity of SOD of *S. calendulacea* also increases on day-12 (Fig. 8b), and this causes the decrease of MDA and relieves the damage of the cell membrane (Fig. 8a), finally results in the increased Pn.

The hybrid between *S. trilobata* and *S. calendulacea* may not expand their geographical range in the short term but the hybridization may accelerate the extinction of its native parent *S. calendulacea* in subtropical China. Many hybrids between native and invasive species evolve and expand their distributions beyond their site of origin^3^,^10^. In this study we found all detected indicators demonstrated that hybridization just slightly catalyzed the tolerance of the hybrid to low temperature and low light condition, but the tolerance was not significantly different from the invasive parent *S. trilobata.* Considering that temperature and light are the major factors limiting the distribution region of *S. trilobata*, the hybrid may not expand its geographical range in the near future. However, introgression is another important method for indirect evolution. Generally, the backcross can enhance the invasiveness of exotic species^9^,^12^. Of concern is that the minor evolved hybrid of *S. trilobata* × *S. calendulacea* backcross with their parents will further enhance its invasiveness and adaptability. Therefore, whether or not the backcrossed hybrid will expand their distribution is still uncertain. But it is certain that hybridization between an invasive species and its native congener, with or without introgression, frequently threatens native populations in a wide variety of plants. It is an important cause of habitat loss for native flora, in addition to habitat destruction and fragmentation^12^. Hybridization can lead to the adaptive evolution of a new hybrid in a number of ways, such as evolutionary novelty, genetic variation, fixed heterosis, and dumping genetic load^9^. In recent decades, the population of the Chinese native species *S. calendulacea* has decreased sharply and the decrease is closely related to the invasion of *S. trilobata*^19^. The new hybrid of *S. trilobata* × *S. calendulacea* may have an equal or even greater adaptative response than its invasive parent *S. trilobata*^19^,^24^. Furthermore, during hybridization between *S. trilobata* and *S. calendulacea*, all maternal parents are the native species *S. calendulacea*^19^. This will increase the reproductive cost to the *S. calendulacea* population. Generally, hybridization produces an abundance of sterile progeny and it will further accelerate the genetic dilution of *S. calendulacea*, finally decreasing the genetic diversity of the species and leading to its extinction^28^,^29^. Therefore, the threat of hybrids to the extinction of its native parent *S. calendulacea* should be acknowledged and steps should be taken to protect the species.

Our research also highlights one of the risks of translocations in ecological restoration^30^. It is sometimes difficult to obtain native plants to restore damaged habitat with the same species or the same genetic population for a species so there are at times substitutions. For example, when restoring degraded wetlands in western North America with clonal graminoid cattails, nurseries may only have the eastern narrowleaf cattail (*T. angustifolia*) in stock and these would be planted rather than the native western broadleaf cattail (*T. latifolia*). Once planted, the narrowleaf cattail hybridizes with the native broadleaf cattail in the wild, producing plants that are taller than their parents and which aggressively overtake the vegetation of a marsh^31^. The narrowleaf cattail itself was initially believed to have been introduced from Europe^32^, but recent studies suggest that it was present in North America prior to European settlement in some areas^33^. Another aggressive wetland hybrid in North America is the perennial grass, Phragmites australis^34^.

In addition to invasiveness, ecological restoration is also susceptible to the problem of genetic dilution although more from intraspecific rather than interspecific breeding. This highlights the importance of the provenance of the plant stock used to restore habitat. Studies such as those on the wooly yarrow (*Achillea lanulosa*) by pioneers of ecological genetics long ago established the importance of matching genetic stock to geographic location for many species^35^. Commonly applied guidelines on public lands in the United States suggest that plant material for out-planting be collected within 1,000 ft (305 m) of elevation and 100 miles lateral distance^36^. Although there is no clear answer on how close geographically plant stock needs to be collected for a restoration project^37^,^38^, the assumption is that, according to the distance decay of similarity principle^39^, populations growing nearer to each other are closer genetically. Genetic similarity in ecological restoration is especially important for species at risk. Source material for many of the endangered meadow wildflowers of the Garry Oak Ecosystem that reproduce vegetatively is often collected within 20 km of a restoration site^40^. Conversely, species that are wind pollinated or seed dispersed have larger effective populations so plant stock can be collected for out-planting from over a much larger area, perhaps hundreds of kilometres.

## Material and Methods

### Plant Materials and Experimental Design

The distributions of *S. trilobata*, *S. calendulacea* and their hybrid usually form a mosaic along riverbanks, coastlands and other moist habitats in subtropical China. This region has a subtropical monsoon humid climate with distinct wet (April to September) and dry (October to March) seasons. The LTWL condition usually occurs in winter during the dry season and sometimes it can limit plant distribution. In this study, plant material was collected from South China Botanical Garden, Chinese Academy of Sciences, Guangzhou, China (23°10′N, 113°21′E). *S. trilobata*, *S. calendulacea* and their hybrid were found around two lakes in the South China Botanical Garden^24^. Collected plant material was cut into 10 cm lengths (with two internodes) and then planted in a bed of sand. After two weeks cultivation in a low light environment, the homogeneous seedlings of each species were selected and transplanted into plastic pots that were filled with equal proportions of river sand and pool mud. Hereafter, the plastic pots were cultivated for 30 days in a greenhouse in South China Normal University. Then 10 plastic pots of each species were transferred to a constant temperature and humidity incubator and cultivated for 20 days. The temperature was set as 4 °C, with a photoperiod of 16 h; relative humidity was ca. 65% and photosynthetic photon flux density (PPFD) was ca. 200 μmol m^−2^ s^−1^ during the day. Four pots of each species were selected to measure their gas exchange parameters and chlorophyll fluorescence parameters. In order to ensure there were enough leaves for the measurement, the other six pots of each species were prepared to measure the content of MDA and the activity of SOD and POD, although only four of these six individuals were measured every time. At the same time, the phenotype, gas exchange parameters, chlorophyll fluorescence parameters, content of MDA and activity of SOD and POD were first recorded and measured before treatment. After 16 days of treatment, all parameters mentioned above were measured again.

### Phenotypic Change

The phenotypic changes of the experimental materials were recorded every 4 days using a digital camera (D7000, Nikon, Japan).

### Gas Exchange Parameters

The net photosynthetic rate (Pn), stomatal conductance (Gs), and transpiration rate (Tr) of *S. trilobata*, *S. calendulacea* and their hybrid were measured using a programmable, open-flow gas exchange system (Li-6400, Li-Cor, USA) before and after 16 days of treatment. During each measurement, the gas exchange parameters were measured on four fully expanded young leaves (the 3rd–5th new leaves), from different individuals per species from 08:00 to 10:00 in the morning. Leaf chamber environmental conditions were set to 26 °C and 200 μmol m^−2^ s^−1^ PPFD.

### Chlorophyll fluorescence parameters

The chlorophyll fluorescence parameters, including the maximal photochemical efficiency of PS II (F_v_/F_m_), the quantum yield of PS II (Yield), the effective photochemical efficiency of PS II (Φ_PS II_) and the total electron transport rate through PS II (ETR), were measured with a portable pulse-modulated fluorimeter PAM-2100 (Walz, Efeltrich, Germany). Individuals were kept in a dark environment for 30 min before measuring the minimum (F_0_) and maximum fluorescence (F_m_). F_v_/F_m_ was calculated as F_v_/F_m_ = (F_m_ – F_0_)/F_m_^41^. After the light-adaption under an actinic light irradiance of 200 μmol m^−2^ s^−1^, the steady-state (F_s_) and maximum fluorescence (F_m_’) were measured. Φ_PS II_ was calculated as Φ_PS II_ = (F_m_’ − F_s_)/F_m_’^42^. ETR was estimated according to Krall and Edwards^43^: ETR = Φ_PS II_ × PPFD × A × 0.5, where A is the leaf absorptance, which was estimated as 0.84. The factor 0.5 was based on the assumption of an equal distribution of photons between PS I and PS II. Partitioning of the absorbed light energy was calculated referring to the model built by Hendrickson *et al.*^44^. The quantum yield of PS II was estimated as Yield = 1–(F_s_/F_m_’). The quantum efficiency of regulated △pH- and/or xanthophyll-dependent non-photochemical dissipation processes within the PS II antennae (Φ_NPQ_) was calculated as: Φ_NPQ_ = F_s_/F_m_’ − F_s_/F_m_. Constitutive non-photochemical energy dissipation and fluorescence was calculated as: Φ_f,D_ = F_s_/F_m_^45^.

### MDA, SOD and POD

The content of malonaldehyde (MDA) was measured by thiobarbituric acid (TBA) reaction to estimate the degree of lipid peroxidation in leaf tissue. Leaves (0.1 g, four repeats each species from different individuals), were ground and homogenized with 3 mL of 50 mmol/L phosphate buffer solution (PBS, pH = 7.8). The homogenate was centrifuged at 3,500 × g for 15 min. Then 2 mL of 20% TCA containing 0.5% (*w*/*v*) TBA were added in the supernatant liquor. The mixture was heated at 98 °C for 20 min and then quickly cooled in ice water. The contents were centrifuged at 10,000 × g for 15 min, and the absorbance was measured at 532 nm. The value of nonspecific absorption at 600 nm was subtracted. The concentration of MDA was calculated using an extinction coefficient of 155 mM cm^−1^. Results were expressed as micromoles per gram FW.

SOD was measured by the NBT method modified from Beyer and Fridovich^46^. Leaves (0.1 g, four repeats for each species from different individuals) were ground and homogenized with 2 mL of extracting solution (50 mmol/L PBS (pH = 7.8), 0.1 M EDTA, 0.1% (v/v) Triton X-100, 2% (w/v) PVP) on ice. The homogenate was centrifuged at 3,500 × g for 15 min. Then 0.1 mL of the supernatant liquor was added into the reaction system (2 mL of 50 mmol/L PBS, 0.3 mL of 130 mmol/L Met, 0.3 mL of 750 μmoL/L NBT, 0.3 μL of 0.1 moL/L Na_2_-EDTA, 0.3 mL of 20 μmoL/L riboflavin). The reaction was started by the addition of Met and riboflavin. The reaction tubes were placed beside a set of 4000 lx fluorescent tubes for 15 min. Then the reaction system was measured with a spectrophotometer at 560 nm. One unit of SOD was defined as the amount contained in the volume of extract that caused a 50% inhibition of the SOD-inhibitable fraction of the NBT reduction.

POD activity was measured according to Ramanathan *et al.*^47^ with some modification. Enzyme extracts for each sample were taken (0.1 mL) then reacted with 0.025 mL guaiacol (50 mmol/L) in 1.875 mL phosphate buffer (50 mmol/L, pH = 7.0) and 1 mL hydrogen peroxide (30 mmol/L) as an initiator. Directly after adding the initiator, enzyme activity was measured as change of absorbance at wave length 470 nm for 3 min at room temperature. POD activity was expressed as the change of U/min/g fresh leaves. One activity unit (U) of POD was defined as the activity that caused a 0.01 increment of absorbance per minute.

### Statistical Analysis

Data between untreated and treated samples after 16 days were not totally independent from each other. The data were analyzed using a two-way ANOVA suitable for data with one repeated measurement (type of matrix = UN, P = 0.05). Data between different species measured at the same time were analyzed using a one-way ANOVA (P = 0.05). Data were presented as means ± 1 SD. The analysis was carried out using SAS for Windows V8.

## Additional Information

**How to cite this article**: Sun, Z. *et al.* Responses of the Hybrid between *Sphagneticola trilobata* and *Sphagneticola calendulacea* to Low Temperature and Weak Light Characteristic in South China. *Sci. Rep.* **5**, 16906; doi: 10.1038/srep16906 (2015).
